# Intraocular pressure-lowering effect of *Cordyceps cicadae* mycelia extract in a glaucoma rat model

**DOI:** 10.7150/ijms.47912

**Published:** 2021-01-01

**Authors:** Chi-Ting Horng, Ya-Lan Yang, Chin-Chu Chen, Yu-Syuan Huang, Chun Chen, Fu-An Chen

**Affiliations:** 1 Department of Ophthalmology, Fooyin University Hospital, Pingtung, Taiwan; 2 Department of Pharmacy and Master Program, Tajen University, Pingtung, Taiwan; 3 Grape King Bio Ltd, Zhong-Li Dist., Taoyuan City, Taiwan

**Keywords:** *Cordyceps cicadae* mycelia, Glaucoma, Intraocular pressure, Antioxidant

## Abstract

Glaucoma is a leading cause of irreversible blindness worldwide. This study evaluates the reduction of intraocular pressure (IOP) induced by *C. cicadae* mycelia extract in a steroid-induced rat model of glaucoma. *Cordyceps cicadae* mycelia is a well-known and valued traditional Chinese herbal medicine. *C. cicadae* mycelia were cultured using a liquid fermentation technique. The harvested *C. cicadae* mycelia were then lyophilized and extracted with two solvents, water and ethanol. The aqueous extract (CCM-DW) and ethanolic extract (CCM-EtOH) of the mycelia were obtained through lyophilization. Sprague Dawley rats were randomly divided into four groups (n = 6 in each group): a normal group, a control group, and experimental groups treated with CCM-DW, or CCM-EtOH (both at 50 mg/kg/body weight). Except for those in the normal group, all rats received a subconjunctival injection of betamethasone to induce high IOP. The rats in the experimental groups received a daily administration of CCM by oral gavage for four consecutive weeks. IOP reduction is the known treatment for glaucoma. The results revealed that steroid treatment caused a significant increase in the animals' IOP (control group). Elevated IOP decreased significantly after treatment with CCM-DW and CCM-EtOH (p < 0.01), and CCM-DW was more effective than CCM-EtOH. CCM-DW and CCM-EtOH were capable of causing significant decreases in high IOP-induced lesions in pathological studies in which it was shown that the efficacy of CCM-DW surpassed that of CCM-EtOH. After CCM-DW administration for 28 days, there were significant decreases in malondialdehyde and lactate dehydrogenase levels and significant increases in catalase, superoxide dismutase, and glutathione peroxidase levels. In summary, *C. cicadae* mycelia may be beneficial for preventing or treating glaucoma due to its significant IOP-lowering and antioxidant activities.

## Introduction

Glaucoma is one of the leading causes of vision loss worldwide 1, and elevated intraocular pressure (IOP) is a known risk factor for glaucoma and optic nerve damage 2. In 2013, an estimated 64.3 million people worldwide were affected by glaucoma and were at risk for vision loss, including blindness. This number may increase to 76.0 million in 2020 and 111.8 million by 2040 3. High IOP results from the accumulation of aqueous humor in the anterior chamber, which is primarily due to the eye's lack of capabilities to drain aqueous fluid sufficiently. Blood flowing through the arteries of the ciliary body is the primary source of aqueous humor. The aqueous humor in the posterior chamber is secreted by the ciliary body between the iris and lens. The humor then flows from the posterior to the anterior chamber between the cornea and the iris before being drained from the eye at the iridocorneal junction. In a normal eye, the amount of aqueous humor produced is equal to the amount draining out. However, when this mechanism is compromised, elevated IOP occurs 4. Increased IOP is considered a major risk factor for progressive loss of retinal ganglion cells (RGCs) in the retina 5. Research indicates that a longer the duration of IOP elevation correlates with more severe effects on the optic nerve 6. Common treatment options for glaucoma include medications, surgery, and laser therapy 7. Ocular hypotensive drugs can act by decreasing aqueous humor production or improving trabecular meshwork, Schlemm's canal, or uveoscleral outflow 8. However, most clinical drugs have potential side effects, and natural plant extracts could provide alternative sources of medicine.

Herbal medicines have become a prominent global field of research for health care. *C. cicadae* has long been used as a Chinese herbal medicine since ancient times. *C. cicadae* belongs to the Clavicipitaceae family, and is also known as cicadae flower or Chan-hua. It parasitizes the larvae or nymphs of cicadas (*Cicada flammate*) and forms a biological complex of larva and fungus 9-11. The Compendium of Materia Medica notes that *C. cicadae* is a valuable traditional Chinese herbal medicine for the treatment of epilepsy in children, palpitations, eye diseases, and nighttime crying. Several studies have also reported that *C. cicadae* has a wide variety of pharmaceutical properties and applications, including lipid metabolism regulation, immune regulation, renal function and vision improvements, as well as antitumor, anti-fatigue, anti-inflammatory, and antidiabetes properties 11-19. In addition, the varous constitents of *C. cicadae* include polysaccharides, nucleosides, cordycepins, N6-(2-hydroxyethyl) adenosine (HEA), cyclic heptapeptide, mannitol, and ergosterol 16. Based on the diversity of its composition and efficacy, *C. cicadae* has become a focus in recent research. Thus far, however, relatively little scientific literature has addressed the beneficial effects of *C. cicadae* on patients with glaucoma. Therefore, this study evaluates the effects of *C. cicadae* mycelia on IOP in a rat model of glaucoma.

## Materials and methods

*C. cicadae* mycelia were cultured in a Grape King Bio Ltd. factory according to the following procedure. *C. cicadae* mycelia grown on potato dextrose agar were transferred to 1 L of basal medium (2% glucose, 1% yeast extract, and 1% yellow bean; pH 6.0) in a 2-L flask and shaken at 120 rpm and 25 °C. Three days later, the mycelial culture was inoculated into a 500-L fermenter containing 400 L of basal medium and stored at 25°C for three days. The submerged mycelial culture was heated at 100 °C for 3 h. Subsequently, *C. cicadae* mycelia powder was obtained through lyophilization. The freeze-dried powder was further extracted with distilled water and ethanol. The extracted solutions were then filtered, concentrated, lyophilized, and ground to obtain extract powder (referred to as “CCM” hereafter).

### Phytochemical and antioxidant analysis of *C. cicadae* mycelia extract

A phytochemical analysis was carried out on CCM (CCM-DW and CCM-EtOH), including HEA (C_12_H_17_N_5_O_5_), polyphenols, and polysaccharides. HEA was quantified according to a previous study 17. The content of polyphenols in CCM was measured spectrophotometrically using Folin-Ciocalteu reagent based on a colorimetric oxidation/reduction reaction and was expressed as micrograms of gallic acid equivalent (GAE) 20, 21. The polysaccharide content in CCM was estimated using the phenol-sulfuric acid method and expressed as galactose equivalents (GE) 22, 23. The antioxidant activity of CCM was analyzed using the 2, 2-azinobis (3-ethylbenzthiazoline-6-sulphonate (ABTS) radical scavenging assay 24, 25. The antioxidant activity of CCM was calculated as the ABTS radical scavenging activity (%) based on cation decolorization of the ABTS radical in the presence of CCM.

### Glaucoma model in rats

Twenty-four male Sprague Dawley (SD) rats at seven weeks of age were purchased from BioLASCO Taiwan Co., Ltd. (Yilan, Taiwan). The rats were maintained in 12-h dark/light cycles and housed at 22 ± 2 °C for one week prior to the experiment. The animals were provided with clean water ad libitum and a rodent diet. The experimental protocol was approved by the Institutional Animal Care and Use Committee (IACUC) of Tajen University (IACUC Approval No. 106-04) and conducted according to the guidelines of The Association for Research in Vision and Ophthalmology (ARVO). The SD rats used in the study were divided into four groups (n = 6 in each group): a normal group, control group, a group that received CCM-DW and a group that received CCM-EtOH. In all animals, the right eyes were used as the experimental eyes, and the rats' tails were marked with a felt-tipped pen. Except for those in the normal group, all rats received a subconjunctival injection of betamethasone disodium phosphate (10 mg/mL, 0.03 mL) to induce high IOP, which was monitored every 2-3 days using an AccuPen handheld tonometer (Accutome, USA). After injection of steroids, 1-2 drops of an antibiotic, 0.15% moxifloxacin hydrochloride (Vigmox; Alcon, Inc. Bayer, USA), were administered to prevent eye infection. After about 7 days, the elevated IOP varied within the range of 30-40 mmHg in the experimental SD rats, indicating glaucoma. The experimental groups were administered CCM-DW (50 mg/kg/bw/day) and CCM-EtOH (50 mg/kg/bw/day) by gastric gavage. Doses with no toxic effects were selected based on previous studies 26, 27. IOP in the right eyes of the rats was then measured weekly. Four weeks after completion of the experiments, all the rats were sacrificed and subjected to biopsies of all dissected right eyes. Serum biochemical parameters for malondialdehyde (MDA), catalase (CAT), superoxide dismutase (SOD), glutathione (GSH), GSH peroxidase (GPx), hemoglobin (ABTS), and lactate dehydrogenase (LDH) were determined.

### Histopathological evaluation

All eye tissue specimens were immersed in Davidson's Fluid and fixed after 48 h. The fixative was replaced with 10% formalin and subsequently embedded in hydroxyethyl methacrylate. We obtained a series of sections at intervals of 5 μm, which were all stained with hematoxylin and eosin. Images were captured under a light microscope (Nikon, Japan) for histopathological evaluation.

### Statistical analysis

All data are expressed as the mean ± standard deviation (n = 6). Changes in body weight before and after treatment, IOP, and serum biochemical parameters were analyzed by one-way analysis of variance (ANOVA). Differences were evaluated using ANOVA followed by Dunnett's multiple comparison tests, and p < 0.05 was considered statistically significant.

## Results

### Phytochemical and antioxidant analysis of *C. cicadae* mycelia extract

The results of HEA, phytochemical, and antioxidant analyses of CCM are shown in Table 1. The HEA content was 1.6 and 1.2 mg/g in CCM-DW and CCM-EtOH, respectively. The phytochemical compositions for polyphenols in CCM-DW and CCM-EtOH were 45.7 ± 1.0 and 31.6 ± 0.7 mg GAE/g, and the polysaccharide contents were 330.0 ± 2.9 and 50.9 ± 0.9 mg GE/g, respectively. According to the antioxidant analysis, CCM has significant ABTS radical scavenging activity. The ABTS radical scavenging percentages of CCM-DW and CCM-EtOH were 74.5% and 49.0% at a concentration of 2.5 mg/ml, respectively.

### Effect of *C. cicadae* mycelia extract on body weight in the glaucoma animal model

As shown in Table 2, significant average weight loss occurred in the groups with high IOP at the time of glaucoma induction, but not the normal group. Loss of appetite may have led to weight loss in animal groups with high IOP. Compared with the control group, a trend toward an increase in average body weight was observed for the groups that received CCM for four weeks.

### Effects of *C. cicadae* mycelia extract on IOP in the glaucoma animal model

**Fig 1:**
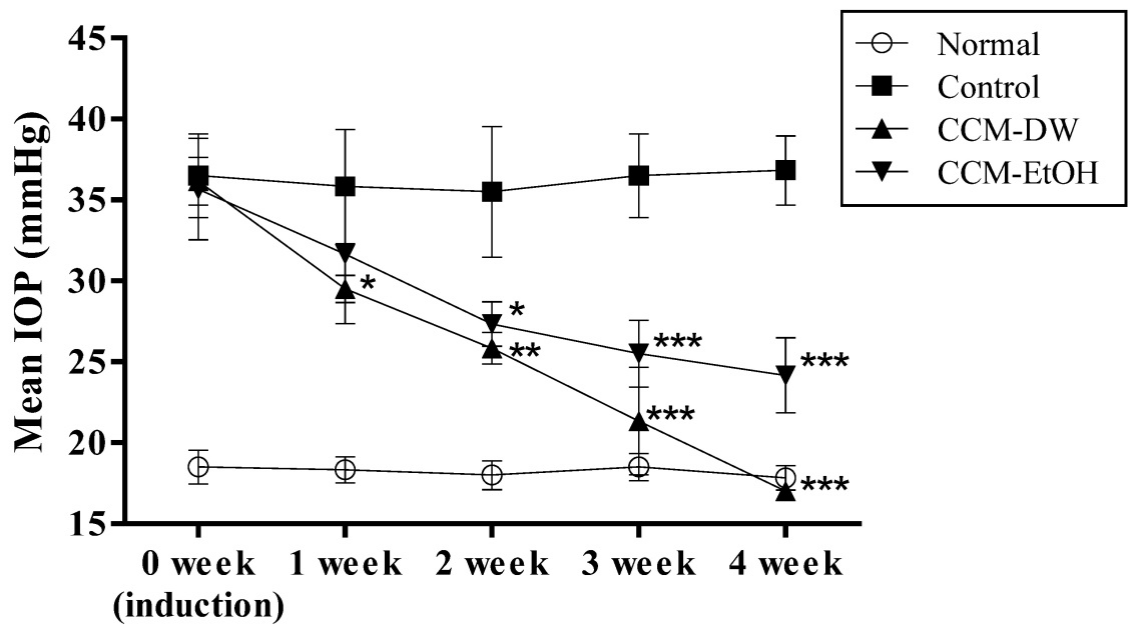
Effect of *C. cicadae* mycelia extract on IOP in the glaucoma model. Data are expressed as mean ± standard deviation (n = 6). *p < 0.05, **p < 0.01, and ***p < 0.001 indicate a significant difference from the control group. CCM-DW (*C. cicadae* mycelia aqueous extract, 50mg/kg/bw/day); CCM-EtOH (*C. cicadae* mycelia ethanolic extract, 50 mg/kg/bw/day).

The effects of CCM on IOP in the glaucoma animal model are shown in Fig. 1. The IOP of the normal and the control groups at 0 week were 18.2 ± 0.3 and 36.9 ± 1.0 mmHg, respectively. The results revealed that the steroid-treated groups experienced significantly increased IOP at 0 weeks after steroid induction compared with the normal group, indicating that the glaucoma animal model with high IOP was successfully induced. The IOP levels after oral administration of CCM-DW and CCM-EtOH for four consecutive weeks were 17.5 ± 0.5 and 24.2 ± 2.3 mmHg, respectively. The experimental results demonstrate that the CCM-DW and CCM-EtOH groups experienced significant reductions in elevated IOP induced by steroids (p < 0.01) in a time-dependent manner over the course of four weeks, and CCM-DW exhibited superior efficacy for IOP reduction. Notably, IOP decreased to its normal range in the CCM-DW group after four weeks of treatment.

### Effects of *C. cicadae* mycelia extract on serum biological parameters in the glaucoma animal model

The effects of the extracts on serum biological parameters in the glaucoma animal model are shown in Table 3. In the control group, the seum levels of MDA and LDH significantly increased (p < 0.05), whereas those of CAT, SOD, and GPx significantly decreased (p < 0.05) compared with the normal group. CCM-DW led to significant decreases in MDA and LDH levels and significant increases in CAT, GPx, and SOD levels after oral administration of CCM-DW for four weeks (p < 0.05). CCM-EtOH exhibited a similar trend in results as those of CCM-DW but not at significant levels. The experimental results indicated that CCM-DW was capable of enhancing the antioxidant capacity in the glaucoma rats.

### Effects of *C. cicadae* mycelia extract on optical pathological observations in the glaucoma animal model

**Fig 2:**
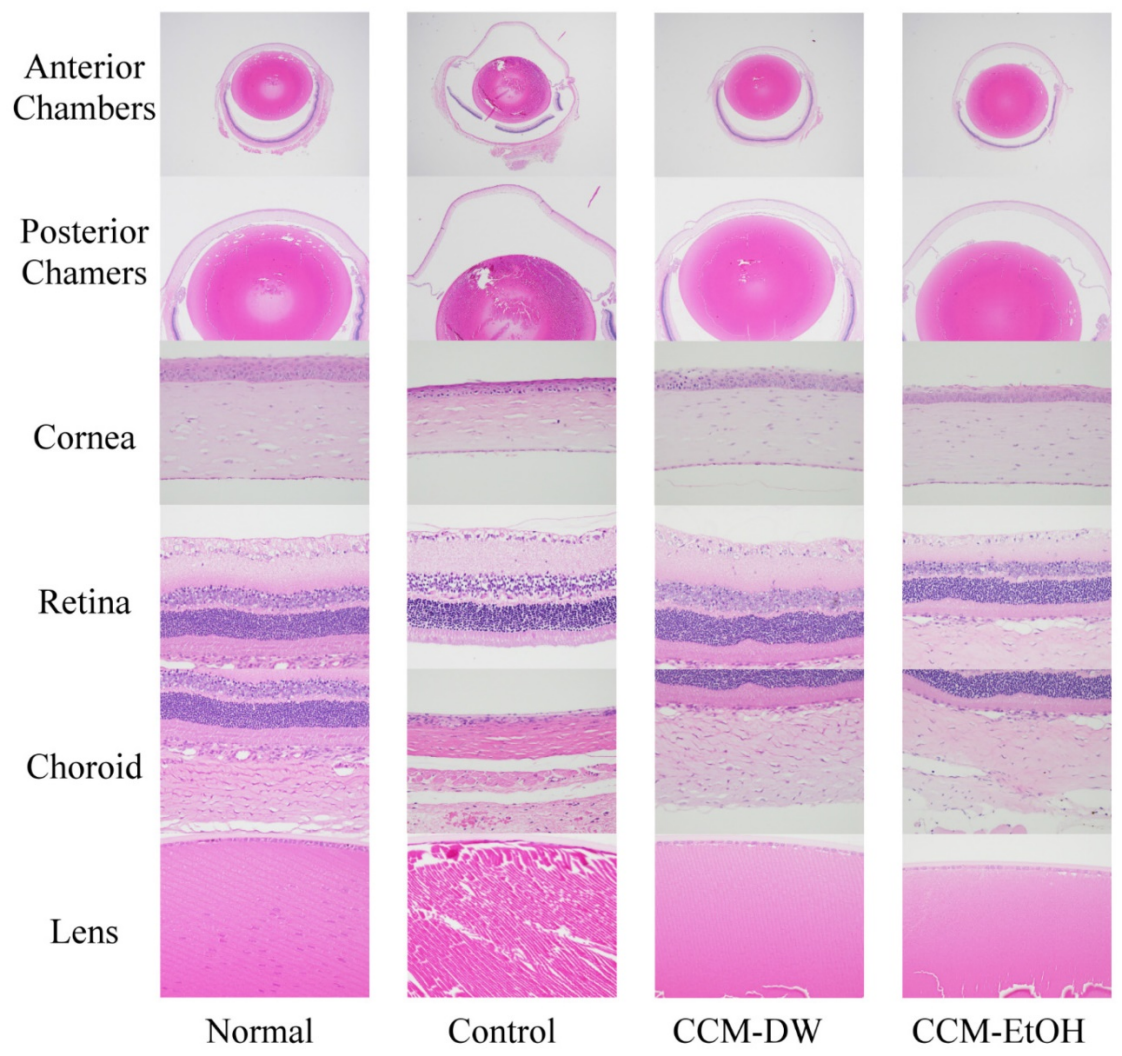
Histopathological findings for eyes in the chronic intraocular hypertension model. Anterior and posterior chambers with vitreous humor (20×, 40×), cornea (400×), retina (400×), choroid (400×), and lens (400×). Haematoxylin and eosin stain.

The histopathological findings for normal and chronic ocular hypertension eyes are presented in Fig. 2. The normal group exhibited whole eyes with normal anterior and posterior chambers, vitreous humor, cornea, retina, choroid, and lens. Glaucoma eyes exhibited moderate dilation in the anterior and posterior chambers with vitreous humor dilation, retinal detachment, and thin cornea, retina, and choroid, in addition to lens degeneration in the control group. The CCM-DW group exhibited whole eyes with normal anterior and posterior chambers and vitreous humor, cornea, retina, choroid, and lens. Whole eyes with slight anterior and posterior chambers with vitreous humor, normal cornea, retina, choroid, and lens were observed in the CCM-EtOH group. Regarding the micro-findings of each group, lesions were graded from 1 to 5 for severity: (1) minimal (<1%), (2) slight (1%-25%), (3) moderate (26%-50%), (4) moderate-severe (51%-75%), and (5) severe or high (76%-100%). As shown in Table 4, the normal group showed no lesions in the anterior and posterior chambers, vitreous humor, cornea, choroid or sclera, as well as no retinal detachment, no retinal thinning, and no ciliary bodies. This group had a score of 0 ± 0. In the control group, these items yielded lesion scores of 1.3-4.5, indicating that high IOP caused slight to severe damage (p < 0.01). No obvious lesions were observed in the CCM-DW group, which scored 0.0 ± 0.0, whereas the CCM-EtOH group experienced minimal lesions in the anterior and posterior chambers with scores of 0.7 ± 1.0. These results indicate that CCM-DW and CCM-EtOH were capable of significantly minimizing lesions caused by high IOP in glaucoma rats according to pathological observations (p < 0.01), and the efficacy of CCM-DW was superior to that of CCM- EtOH.

## Discussion

Glaucoma is the second most common cause of blindness worldwide and is characterized by progressive degeneration of the optic nerve and RGCs 28. Although other factors have been proposed as playing roles in glaucoma, high IOP remains the primary documented risk factor for glaucomatous optic nerve damage 29. For the treatment of eye disease, following the administration of benzalkonium chloride, fermented mycelia extracts of *C. cicadae* reportedly enhanced corneal resilience and the maintenance of conjunctival goblet cells, thus causing an improvement in dry-eye symptoms 27. In the present study, we first evaluated the IOP-lowering effects of *C. cicadae* mycelia extract in a steroid-induced glaucoma rat model for glaucoma disease treatment.

The results revealed that steroid treatment-induced glaucoma led to a significant increase in IOP and decrease in the average weight of rats. Steroid-induced elevated IOP is reportedly dose-dependent and followed by known systemic toxic effects, including weight loss 30. Even though the common side effects of glucocorticoid in humans include weight gain 31, the development of stomach irritation is also commonly observed, which could lead to the loss of appetite and weight in rats. However, body weight loss improved slightly compared with the control group after the administration of CCM for four weeks. Compared with the normal group, IOP significantly increased by approximately 20 mmHg in the groups given steroid (subconjunctival betamethasone injection), indicating that the glaucoma animal model was successfully induced. The experimental results revealed that CCM-DW and CCM-EtOH were capable of significantly lowering IOP in glaucoma rats (p < 0.01) in a week-dependent manner, and the effectiveness of CCM-DW was higher than that of CCM-EtOH. In the CCM-DW group, IOP was restored to its normal range after four weeks of treatment compared with the normal group. Pathological observations revealed significant lesions for the anterior and posterior chambers, vitreous humor, cornea, choroid, and sclera in addition to retinal detachment, retinal thinning, and ciliary bodies in the control group. No evident lesions were observed in the micropathological findings of the CCM-DW group, whereas the CCM-EtOH group exhibited minimal-grade lesions in the anterior and posterior chambers. The results indicated that CCM-DW and CCM-EtOH caused a significant decrease in IOP-induced lesions in glaucoma rats according to histopathological findings, and the efficacy of CCM-DW was superior to that of CCM-ETOH. The control group had significantly increased MDA and LDH levels and significantly decreased GPx, CAT, and SOD levels, indicating that the glaucoma rats had high oxidative stress levels. In the experimental groups, CCM-DW caused a significant decrease in MDA and LDH levels and a significant increase in GPx, CAT, and SOD levels after four weeks of treatment. CCM-EtOH yielded similar trend results. The experimental results indicate that *C. cicadae* mycelia extract enhanced the intrinsic antioxidant capabilities of the glaucoma rats.

Currently, medical therapy to lower IOP remains the most common initial treatment method in addition to surgical procedures and laser treatment for glaucoma. Medical therapy involves two mechanisms to control IOP: enhancing outflow drainage and hindering the production of aqueous humor 32. Among the many drugs available for glaucoma treatment, rho-associated protein kinase (ROCK) inhibitors have commonly been used to alter the shape of trabecular meshwork cells, allowing for enhanced aqueous humor outflow to lower IOP 33. However, according to our unpublished data, the IOP-lowering effects of CCM-DW and CCM-EtOH are not mediated through the ROCK/myosin light chain phosphorylation (MLC) pathway, suggesting that there may be other pathways.

Adenosine and different adenosine receptor subtype agonists (A1, A2, and A3) have also been proposed as potential IOP lowering drugs 34. Previous studies have shown that topical treatment with an adenosine A1 receptor agonist can significantly decrease IOP in monkeys 35, rabbits 36, and mice 37. Moreover, A2 and A3 adenosine receptor agonists are currently being evaluated in larger populations in the clinical trial phase with the expectation of lowering IOP 33. The proposed mechanism of these adenosine receptor agonists is via the induction of the level of matrix metalloproteinases (MMPs), which leads to remodeling of the extracellular matrix in the trabecular meshwork and a consequent increase in aqueous humor outflow. As a derivative of adenosine, HEA may exert pharmacological activity by acting on adenosine receptors. Nevertheless, further studies are needed to clarify this point.

Antioxidant pretreatment reportedly results in a marked reduction of the effect of oxidative stress on the trabecular meshwork 38. *C. cicadae* mycelia has been reported to have a high clearance rate of 2',2'-diphenyl-1-picrahydrazyl (DPPH) free radicals 39. *C. cicadae* has also been reported to have antioxidant components, including polysaccharides, cordycepin, HEA, and ergosterol 16. Intracellular polysaccharides and extracted exopolysaccharides from *C. cicadae* exhibit higher antioxidant potential with significant ABTS and DPPH radical-scavenging activities, reducing power, and iron chelating activity 40. In addition, HEA isolated from *C. cicadae* has antihyperglycemic, anti-inflammatory, and antioxidant effects in diabetic rats and the results suggested that HEA attenuates inflammation and oxidative stress in kidney tissue 41. HEA is an antioxidant compound from *C. cicadae* and has beneficial effects on oxidative stress-related diseases, including diabetes and kidney disease. Our phytochemical analysis results showed that CCM-DW and CCM-EtOH contains HEA, polyphenol, and polysaccharide components. The amounts of HEA, polysaccharides, and polyphenol contents in CCM-DW were significantly higher than those in CCM-EtOH, suggesting a reason why the effectiveness of CCM-DW was higher than that of CCM-EtOH. In addition, both CCM-DW and CCM-EtOH had higher contents of polysaccharides than polyphenols. Notable, that the polysaccharide content was approximately seven times higher than that of polyphenols in CCM-DW. The results indicate that polysaccharides were the abundant constituent in CCM-DW. The ABTS radical-scavenging assay has been widely used to compare the antioxidant ability of plant extracts. In the case of antioxidant activity, the ABTS radical-scavenging activity of CCM-DW was significantly higher than that of CCM-EtOH. The results indicate that when compared with polyphenols, polysaccharides as the main components of CCM-DW dominate the antioxidant activity of CCM. An increasing amount of evidence shows that reactive oxygen species play a key role in the pathogenesis of primary open-angle glaucoma 42. The correlation among the content of antioxidant components, in vitro/in vivo antioxidant capabilities, and IOP-lowering activity suggest that oxidative stress plays a critical role in the development of IOP increase and glaucoma-associated lesions. Theoretically, based on our overall results, CCM and its antioxidant activity could be associated with lowered IOP and dysfunction of the optic nerve and retinal ganglion cells through antioxidant mechanisms. In addition, male and female SD rats that were administered mycelial *C. cicadae* for 90 days in a sub-chronic toxicity study revealed no observable adverse effects at a dose of > 2000 mg/kg, which suggests that *C. cicadae* is safe for use in functional food development 26.

## Conclusions

In conclusion, this study has indicated that *C. cicadae* mycelia could be used as a food supplement with substantial benefits for relieving glaucoma symptoms through significant IOP-lowering and antioxidant activities. Subsequent studies could focus on the investigation of molecular mechanisms underlying the therapeutic effects of *C. cicadae* mycelia associated with glaucoma. *C. cicadae* mycelia could provide an alternative natural plant resource for glaucoma treatment.

## Figures and Tables

**Table 1:** Phytochemical and antioxidant analysis of *C. cicadae* mycelia extract (CCM).

*C. cicadae* mycelia extract	HEA (mg/g)	Polyphenols (mg GAE/g)	Polysaccharides (mg GE/g)	ABTS radical scavenging activity (%) at 2.5 mg/mL
CCM-DW	1.65 ± 0.02	45.7 ± 1.0	330.0 ± 2.9	74.5 ± 1.0
CCM-EtOH	1.21 ± 0.03	31.6 ± 0.7	50.9 ± 0.9	49.0 ± 2.5

CCM-DW (*C. cicadae* mycelia aqueous extract); CCM-EtOH (*C. cicadae* mycelia ethanolic extract); GAE: gallic acid equivalent; GE: galactose equivalent.

**Table 2:** Effect of *C. cicadae* mycelia extract on body weight in the glaucoma animal model.

Groups	Body weight (g) at the beginning	Body weight (g) at time of glaucoma induction	Body weight (g) after treatment for 4 weeks
**Normal**	239.6 ± 4.9	306.5 ± 7.4	416.8 ± 14.6
**Control**	251.7 ± 8.7	198.8 ± 11.8^#^	204.5 ± 40.4^#^
**CCM-DW**	243.1 ± 11.7	193.1 ± 12.2	238.4 ± 53.9
**CCM-EtOH**	243.5 ± 9.7	188.3 ± 17.6	221.9 ± 45.8

Data are reported as mean ± standard deviation (n = 6). CCM-DW (*C. cicadae* mycelia aqueous extract, 50mg/kg/bw/day); CCM-EtOH (*C. cicadae* mycelia ethanolic extract, 50 mg/kg/bw/day.

**Table 3:** Effects of *C. cicadae* mycelia on hepatic and renal serum biological parameters in the glaucoma rat model.

Groups	CAT (U/mg)	GSH (U/mg)	GPx (U/mg)	ABTS (mg/mL)	MDA (nmol/mg)	SOD (U/mg)	LDH (U/L)
**Normal**	57.92±12.66	1.34±0.03	1.05±0.32	19.27±1.88	0.83±0.02	107.14±8.61	1495.7±320.9
**Control**	19.71±11.45^#^	1.32±0.07	0.64±0.27^#^	19.19±2.74	1.31±0.03^#^	80.85±9.16^#^	2167.0±334.1^#^
**CCM-DW**	40.52±14.96^*^	1.55±0.03	1.05±0.37^*^	21.55±1.67	1.01±0.01^*^	112.61±9.67^*^	1294.8±815.1^*^
**CCM-EtOH**	27.06±16.26	1.38±0.03	0.75±0.29	21.18±2.09	1.26±0.03	82.79±2.36	1739.2±798.7

Data are reported as mean ± standard deviation (n = 6). #p < 0.05 compared with the normal group; *p < 0.05 compared with the control group. CAT, catalase; GSH, glutathione; GPx, glutathione peroxidase; ABTS, 2,2'-Azinobis-(3-ethylbenzthiazoline-6-sulphonate); MDA, malondialdehyde; SOD, superoxide dismutase; LDH, lactic dehydrogenase. CCM-DW (*C. cicadae* mycelia aqueous extract, 50mg/kg/bw/day); CCM-EtOH (*C. cicadae* mycelia ethanolic extract, 50 mg/kg/bw/day).

**Table 4:** Effects of *C. cicadae* mycelia on micropathological observations in the glaucoma model.

Groups	anterior chamber, posterior chamber	Vitreous humor	Cornea	Choroid and sclera	Retinal detachment	Retinal thinning	ciliary body
**Normal**	0±0	0±0	0±0	0±0	0±0	0±0	0±0
**Control**	3.2±0.8^##^	3.5±0.5^##^	2.7±0.8^##^	3.5±0.8^##^	4.5±1.2^##^	1.7±0.8^##^	1.3±1.0^##^
**CCM-DW**	0±0^**^	0±0^**^	0±0^**^	0±0^**^	0±0^**^	0±0^**^	0±0^**^
**CCM-EtOH**	0.7±1.0^**^	0±0^**^	0±0^**^	0±0^**^	0±0^**^	0±0^**^	0±0^**^

Data are reported as mean ± standard error of the mean (n = 6). ##p < 0.01 compared with the normal group. **p < 0.01 compared with the control group. Lesions were graded from 1 to 5 depending on severity: 1 = minimal (<1%), 2 = slight (1%-25%), 3 = moderate (26%-50%), 4 = moderate-severe (51%-75%), 5 = severe-high (76%-100%).
